# Mitochondrial Fusion Machinery Specifically Involved in Energy Deprivation-Induced Autophagy

**DOI:** 10.3389/fcell.2020.00221

**Published:** 2020-04-07

**Authors:** Choufei Wu, Weijing Yao, Wenwen Kai, Weikang Liu, Wenlve Wang, Shuzhen Li, Yingcong Chen, Xiaoyong Wu, Liefeng Wang, Ying Li, Jingjing Tong, Jing Qian, Liqin Zhang, Zhi Hong, Cong Yi

**Affiliations:** ^1^Key Laboratory of Vector Biology and Pathogen Control of Zhejiang Province, School of Life Sciences, Huzhou University, Huzhou, China; ^2^Department of Biochemistry, Hepatobiliary and Pancreatic Surgery, The First Affiliated Hospital, Zhejiang University School of Medicine, Hangzhou, China; ^3^Key Laboratory of Prevention and Treatment of Cardiovascular and Cerebrovascular Diseases, Ministry of Education, Gannan Medical University, Ganzhou, China; ^4^School of Life Sciences, Tsinghua University-Peking University Joint Center for Life Sciences, Tsinghua University, Beijing, China; ^5^School of Life Sciences, Central China Normal University, Wuhan, China; ^6^Key Laboratory of Vector Biology and Pathogen Control of Zhejiang Province, School of Nursing and Medicine, Huzhou University, Huzhou, China; ^7^Department of Breast Surgery, The Second Affiliated Hospital, Zhejiang University School of Medicine, Zhejiang University, Hangzhou, China; ^8^ZJU-UoE Institute, Zhejiang University School of Medicine, International Campus, Zhejiang University, Haining, China

**Keywords:** mitochondrial morphology, fusion machinery, mitochondrial respiration, autophagy, glucose starvation

## Abstract

Mitochondria are highly dynamic organelles, which can form a network in cells through fusion, fission, and tubulation. Its morphology is closely related to the function of mitochondria. The damaged mitochondria can be removed by mitophagy. However, the relationship between mitochondrial morphology and non-selective autophagy is not fully understood. We found that mitochondrial fusion machinery, not fission or tubulation machinery, is essential for energy deprivation-induced autophagy. In response to glucose starvation, deletion of mitochondrial fusion proteins severely impaired the association of Atg1/ULK1 with Atg13, and then affected the recruitment of Atg1 and other autophagic proteins to PAS (phagophore assembly site). Furthermore, the deletion of fusion proteins blocks mitochondrial respiration, the binding of Snf1-Mec1, the phosphorylation of Mec1 by Snf1, and the dissociation of Mec1 from mitochondria under prolonged starvation. We propose that mitochondrial fusion machinery regulates energy deprivation-induced autophagy through maintaining mitochondrial respiration.

## Introduction

Autophagy is a highly conserved material degradation pathway from yeast to human. It can degrade long-lived proteins, damaged organelles, aggregates, lipid droplets, and RNA, etc (Ohsumi, 2014). In the process of autophagy, a double-layer membrane structure envelops these substances to form autophagosome. Subsequently, autophagosome enters lysosomes/vacuoles through fusion. Finally, these substances are degraded by acid hydrolase in lysosomes/vacuoles (Shibutani and Yoshimori, 2014). Based on different degradation substrates, autophagy can be classified into non-selective autophagy and selective autophagy. Non-selective autophagy is usually also called as autophagy/macroautophagy, which has no selectivity for degradation substrates. Selective autophagy is mediated by specific autophagy receptors. Currently, selective autophagy includes mitophagy, ER-phagy, pexophagy, and ribophagy, etc (Feng et al., 2014). Dysfunction of autophagy is closely related to the occurrence and development of many diseases that are harmful to human health (Zhao and Zhang, 2019).

Mitochondria are multifunctional organelles, which play a key role in cell activities and development, including ATP synthesis, iron and calcium homeostasis, programmed cell death, ROS generation, and fatty acid β-oxidation (Wu et al., 2016). These functions of mitochondria are closely related to the morphology of mitochondria. In eukaryotes, mitochondria form a dynamic tubular networks of continuous movement and interaction with other organelles. This dynamic network is mainly regulated by mitochondrial fusion, fission, and tubulation machinery (Okamoto and Shaw, 2005). In yeast, mitochondrial fusion machinery is composed of Fzo1, Ugo1, Mgm1, and Pcp1. Fzo1 is the evolutionary conserved GTPase, which locates in the outer membrane of mitochondria. Its N-terminus and C-terminus are both facing the cytoplasm, where N-terminus contains GTPase domain, and C-terminus interacts with Ugo1 protein. Mgm1, a second GTPase required for mitochondrial fusion, which locates in the inner membrane of mitochondria. Ugo1 protein acts as the adaptor to connect Mgm1 and Fzo1. When fusion is blocked, mitochondria are distributed as dots in cells (Westermann, 2008). Mitochondrial fission machinery is composed of Dnm1, Fis1, Caf4, and Mdv1. Dnm1 is also the conserved dynamin-related GTPase essential for mitochondrial fission and inheritance, which is localized on mitochondria by interacting with Mdv1 and Caf4. Fis1 mediates mitochondrial localization of Mdv1 on the outer membrane. When fission is blocked, mitochondria form interconnected networks (Schauss et al., 2006). Mitochondrial tubulation machinery is composed of Mmm1, Mmm2, Mdm10, Mdm12, Mdm20, Mdm31, and Mdm32, which plays an important role in the actin-mitochondria attachment, formation of tubular mitochondria and anchoring of mtDNA nucleoids. When tubulation pathway is impaired, mitochondria are changed to large spheres (Okamoto and Shaw, 2005). In addition, proteins like Mdm33 and Gem1, it is not known what their molecular mechanism is, but their deletion severely affected the morphology of mitochondria (Dimmer et al., 2002; Frederick et al., 2004). The change of mitochondrial morphology is closely related to aging, programmed cell death, autosomal dominant optic atrophy, charcot-marie-tooth neurophathy type 2A, and neuronal cell function (Alexander et al., 2000; Danial and Korsmeyer, 2004; Li et al., 2004; Kijima et al., 2005; Kauppila et al., 2017).

Recently, more and more attention has been paid to research on the relationship between autophagy and mitochondria. Mostly studied is the role and molecular mechanism of mitophagy in the process of mitochondrial quality control (Kanki and Klionsky, 2010; Montava-Garriga and Ganley, 2020). Mitochondrial damage and nitrogen starvation can induce mitophagy (Kanki et al., 2009; Okamoto et al., 2009). In yeast, Atg32, a receptor protein of mitophagy, mediates the removal of damaged mitochondria, and mitochondrial fission machinery facilitates mitophagy (Kanki et al., 2009; Okamoto et al., 2009). Nitrogen starvation-induced autophagy is involved in the determination of mtDNA copy number (Medeiros et al., 2018). Our previous study showed that mitochondrial oxidative respiratory chain participates in energy deficient-induced autophagy (Yi et al., 2017). In mammals, the mitophagy receptors FUNDC1, NIX, PINK1/Parkin, and BNIP3, directly interact with LC3 through different signaling pathways under different mitochondrial stress to transport damaged mitochondria to lysosomes for clearance (Wei et al., 2015). Mitochondria also supply membrane source for autophagosome formation during serum starvation; the disruption of mitochondria/ER can block autophagosome biogenesis (Hailey et al., 2010). However, the relationship of mitochondria morphology and non-selective autophagy is not well understood.

In this study, we found mitochondria fusion machinery is involved in initiation of glucose starvation-induced autophagy, by revealing the strong link between mitochondrial fusion machinery and mitochondrial respiration, the association of Snf1 with Mec1, the phosphorylation of Mec1 by Snf1, the recruitment of Atg1/ULK1 and other autophagic proteins to PAS, and the dissociation of Mec1 from mitochondria under glucose starvation condition. We propose mitochondrial fusion protein regulates energy deprivation-induced autophagy through maintaining mitochondrial respiration.

## Results

### Mitochondrial Fusion Machinery Is Essential for Glucose Starvation-Induced Autophagy

**FIGURE 1:**
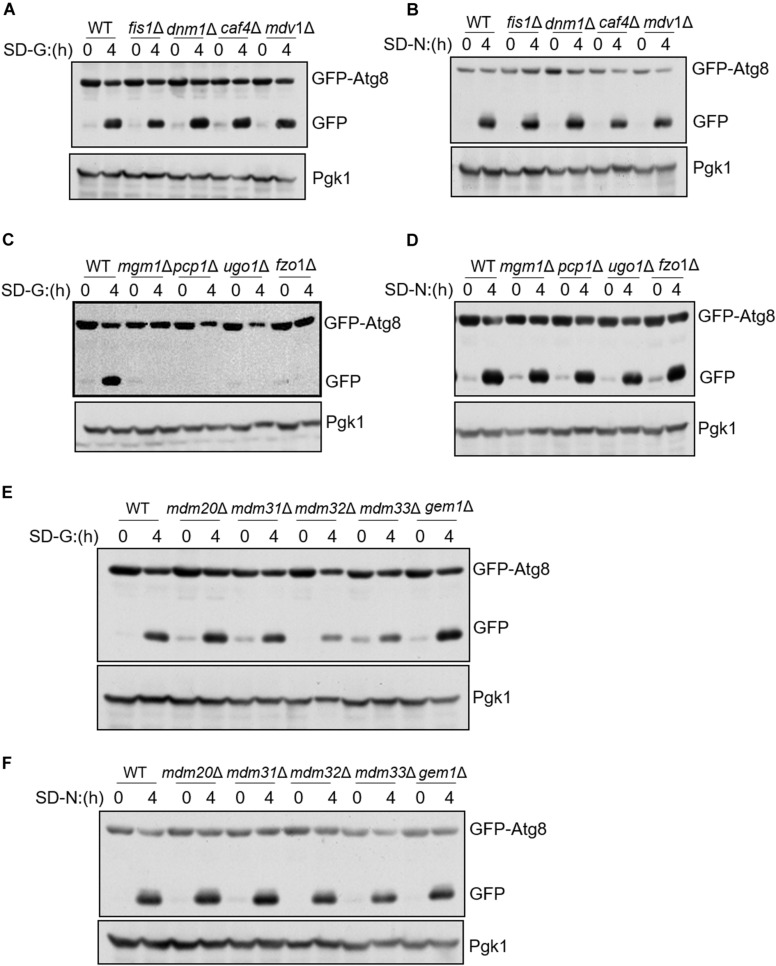
Mitochondrial fusion machinery is specifically involved in glucose starvation-induced autophagy. GFP-Atg8 plasmids were expressed in yeast strains accordingly listed from **(A–F)**. Yeast cells were grown to the log-growth phase, then subjected to glucose starvation (SD-G) or nitrogen starvation (SD-N) for 4 h. Autophagic activity was detected by western blot using anti-GFP antibody.

To know whether mitochondrial fission, fusion, and tubulation machinery are required for autophagy, we knocked out fission/fusion/tubulation genes and analyzed GFP-Atg8 processing in glucose starvation medium (SD-G) and nitrogen starvation medium (SD-N). The principle of GFP-Atg8 assay is that GFP-Atg8 is encapsulated into autophagosome during autophagy process, and then autophagosome fused with vacuole. GFP-Atg8 is degraded by the acid hydrolase in the vacuole, as GFP is relatively stable in acid environment, the efficiency of autophagy can be judged by detecting the amount of free GFP (Sesaki and Jensen, 1999). As shown in Figures 1A,B, we knocked out mitochondrial fission machinery genes *FIS1*, *DNM1*, *CAF4*, and *MDV1*, respectively. Under either nitrogen starvation or glucose starvation, there is no significant difference in the cleavage of GFP-Atg8 compared with wild type, indicating that mitochondrial fission machinery is not involved in autophagy induced by nitrogen starvation and glucose starvation. Subsequently, we deleted the fusion machinery genes of mitochondria: *MGM1*, *PCP1*, *UGO1*, and *FZO1*, GFP-Atg8 processing assay showed that the fusion machinery of mitochondria doesn’t affect the cleavage of GFP-Atg8 under nitrogen starvation, but completely blocked GFP-Atg8 processing under glucose starvation (Figures 1C,D). Furthermore, we knocked out *MDM33*, *GEM1*, and the tubulation machinery genes of mitochondria: *MDM20*, *MDM31*, and *MDM32*, same as mitochondria fission and fusion genes depleted cells, deletion of these genes did not affect nitrogen starvation-induced autophagy. Under glucose starvation, deletion of *MDM32* and *MDM33* slightly impaired the cleavage of GFP-Atg8, while deletion of other genes has no effect on glucose starvation-induced autophagy (Figures 1E,F). Together, these results indicated that mitochondrial fusion machinery is specifically essential for glucose starvation-induced autophagy.

### Mitochondrial Fusion Machinery Regulates the Recruitment of Atg1 and Other Autophagic Proteins to PAS and the Association of Atg1 With Atg13 Under Glucose Starvation

**FIGURE 2:**
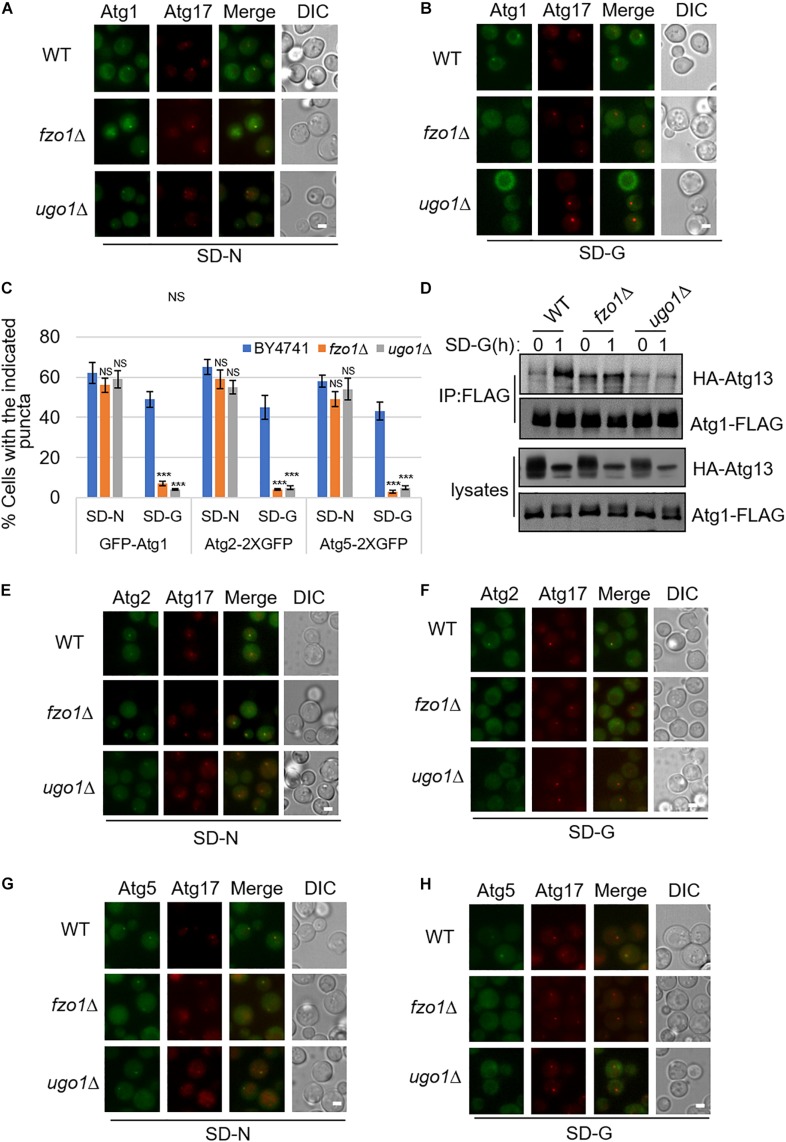
The recruitment of Atg1, Atg2, and Atg5 to PAS is regulated by mitochondrial fusion machinery upon glucose starvation. **(A,B)** GFP-Atg1 and Atg17-2XCherry were co-expressed in wild type (WT), *fzo1*Δ, and *ugo1*Δ yeast strains. Yeast cells were grown to the log-growth phase, then subjected to SD-N and SD-G for 1 h. Cells were imaged by fluorescence inverted microscope. Scale bar, 2 μm. **(C)** Strains from **(A**,**B**,**E–H)** were analyzed for the number of cells with the indicated puncta. *n* = 300 cells pooled from three independent experiments. Data are presented as means ± SD. ***p < 0.001; NS, not significant; two-tailed Student’s *t*-tests were used. **(D)** Wild type (WT), *fzo1*Δ, and *ugo1*Δ yeast cells co-expressing HA-Atg13 and Atg1-3XFLAG were subjected to SD-G for 0 and 1 h. Cell lysates were immunoprecipitated with anti-FLAG agarose beads and then analyzed by western blot using anti-HA antibody. **(E–H)** Atg2-2XGFP or Atg5-2XGFP and Atg17-2XCherry were co-expressed in the wild type (WT), *fzo1*Δ, and *ugo1*Δ yeast strains. Yeast cells were grown to the log-growth phase, then were starved in SD-N and SD-G medium for 1 h. Cells were viewed by fluorescence inverted microscope. Scale bar, 2 μm.

Next, to study which steps during autophagy is mitochondria fusion machinery involved upon glucose starvation, we knocked out *FZO1* and *UGO1* genes, respectively, in yeast strain co-expressing PAS marker Atg17-2XCherry and other autophagy-related proteins labeled with 2XGFP. In yeast cells, Atg17/FIP200, Atg31, and Atg29 proteins form a stable complex independent of nutritional status. Atg1, Atg11, and Atg13 form polymer complex with Atg17-Atg31-Atg29 as a platform for the recruitment of other ATG proteins (Araki et al., 2017). As shown in Supplementary Figures S1A,B, under nitrogen starvation and glucose starvation, Atg17 protein appears as puncta in the *fzo1*Δ and *ugo1*Δ yeast strain, indicating that mitochondrial fusion machinery is not involved in PAS formation. Consistently, Atg11 and Atg13 proteins form puncta and co-localize with Atg17 protein in *fzo1*Δ and *ugo1*Δ yeast strains upon glucose starvation (Supplementary Figures S1A–E). Next, we tested the localization of Atg1 protein. Image data showed that the localization of Atg1 protein dispersed well in the *fzo1*Δ and *ugo1*Δ yeast strains upon glucose starvation. In contrast, Atg1 proteins retained puncta formation despite of *FZO1* and *UGO1* knockout in response to nitrogen starvation (Figures 2A–C). Furthermore, immunoprecipitation experiments also showed glucose starvation did not increase the association of Atg1 with Atg13 in *fzo1*Δ and *ugo1*Δ yeast strains (Figure 2D). Using the same approach, Atg2 and Atg5 became diffused in the *fzo1*Δ and *ugo1*Δ yeast strains upon glucose starvation while remained unaltered upon nitrogen starvation (Figures 2C,E–H). Thus, we concluded that mitochondrial fusion machinery is involved in the recruitment of Atg1 protein to PAS, thus enhancing the recruitment of other proteins to PAS to initiate glucose starvation-induced autophagy.

### Mitochondrial Fusion Proteins Are Required for Mitochondria Respiration and Snf1-Mec1 Association

**FIGURE 3:**
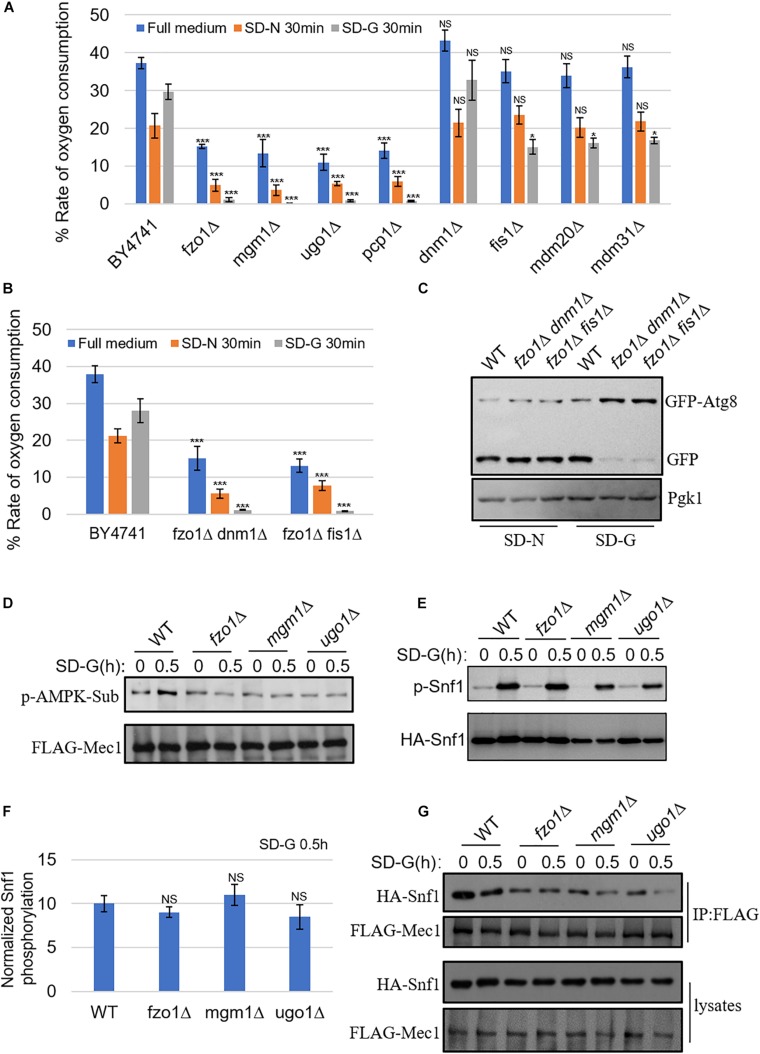
Mitochondrial fusion machinery regulates aerobic respiration of mitochondria and the association of Mec1 with Snf1 upon glucose starvation. **(A)** WT (BY4741), *fzo1*Δ, *mgm1*Δ, *ugo1*Δ, *pcp1*Δ, *dnm1*Δ, *fis1*Δ, *mdm20*Δ, *and mdm31*Δ strains were cultured in SD-N or SD-G for 0 and 30 min. Cells were harvested and oxygen consumption was measured using O2K. *n* = 3 independent experiments were quantified. Data are presented as means ± SD. ***p < 0.001; *p < 0.05; NS, not significant; two-tailed Student’s *t*-tests were used. **(B)** WT (BY4741), *fzo1*Δ *dnm1*Δ, and *fzo1*Δ *fis1*Δ strains were starved in SD-N or SD-G for 0 and 30 min. Cells were harvested and oxygen consumption rate was measured using O2K. *n* = 3 independent experiments were quantified. Data are presented as means ± SD. ***p < 0.001; two-tailed Student’s *t*-tests were used. **(C)** GFP-Atg8 plasmids were expressed in WT (BY4741), *fzo1*Δ *dnm1*Δ, and *fzo1*Δ *fis1*Δ strains. Cells were subjected to SD-G or SD-N for 4 h. Autophagic activity was detected by western blot using anti-GFP antibody. **(D)** FLAG-Mec1 was expressed in the yeast strains listed from Wild type (WT), *fzo1*Δ, *mgm1*Δ *and ugo1*Δ. Yeast cells were grown to the log-growth phase, then subjected to glucose starvation (SD-G) for 0 and 0.5 h. Phosphorylation of immunoprecipitated FLAG-Mec1 was detected by immunoblotting with phospho-(Ser/Thr) AMPK substrate antibody. **(E)** HA-Snf1 was expressed in Wild type (WT), *fzo1*Δ, *mgm1*Δ, and *ugo1*Δ yeast strains. Yeast cells were cultured in glucose starvation (SD-G) for 0 and 0.5 h. The kinase activity of Snf1 was detected by immunoblotting with anti-p-PRKAA/AMPKα (Thr172) antibody. **(F)** Quantification of the ratio of p-Snf1/HA-Snf1 from **(E)** by ImageJ software. NS, not significant; two-tailed Student’s *t*-tests were used. **(G)** FLAG-Mec1 and HA-Snf1 were co-expressed in Wild type (WT), *fzo1*Δ, *mgm1*Δ and *ugo1*Δ yeast strains. Yeast cells were cultured in glucose starvation (SD-G) for 0 and 0.5 h. Cell lysates were immunoprecipitated with anti-FLAG agarose beads and then analyzed by western blot using the indicated antibody.

Our previous report that mitochondrial oxidative respiratory chain-related gene depletion impairs the binding of Atg1–Atg13 through mediating phosphorylation of Mec1 by Snf1 under energy deficiency condition, prompts us to test whether mitochondrial fusion machinery is also involved in the regulation of mitochondrial respiration (Yi et al., 2017). We next performed in-depth bioenergetic studies of mitochondria using O2K for real-time monitoring of mitochondrial oxygen consumption in mitochondrion fusion, fission and tubulation machinery genes deleted yeast strains. As shown in Figure 3A, the aerobic respiration kept at a high level in wild-type yeast cells under full medium, while decreased but remained at a certain level upon nitrogen and glucose starvation. However, in *fzo1*Δ, *mgm1*Δ, *ugo1*Δ, and *pcp1*Δ yeast strains, which mitochondrial fusion is defected, the aerobic respiration decreased significantly under full medium, and almost completely abolished upon glucose starvation. In mitochondrial fission and tubulation-related genes deleted yeast strains, the oxygen consumption rate of mitochondria under full medium and nitrogen starvation condition had no significant change compared with wild type, while the oxygen consumption rate under glucose starvation decreased, but maintained a certain rate. These data suggested that mitochondrial fusion machinery is involved in the regulation of mitochondrial respiration.

To investigate whether the absence of fusion machinery leads to the loss of aerobic respiration by changing mitochondrial morphology, we made fission and fusion double mutant for analysis. Previous study reported that depletion of both fission and fusion genes can rescue the mitochondrial morphological changes caused by the deletion of fusion machinery genes (Sesaki and Jensen, 1999). Consistent with previous study, our image data showed that in the double knockout yeast strains, *dnm1*Δ *fzo1*Δ and *fis1*Δ *fzo1*Δ, mitochondria can be restored to a wild type-like morphology (Supplementary Figure S2). Subsequently, we analyzed mitochondrial oxygen consumption rate, and found that it was completely suppressed in *dnm1*Δ *fzo1*Δ and *fis1*Δ *fzo1*Δ mutants under glucose starvation (Figure 3B). We also tested whether glucose starvation-induced autophagy is affected in double knockout strains. As shown in Figure 3C, glucose starvation-induced autophagy was blocked in these mutants. Thus, we concluded that the inhibition of mitochondrial aerobic respiration is not due to the morphological changes of mitochondria caused by the absence of fusion machinery.

Next, we tested whether Snf1-phosphorylated Mec1 was affected by the absence of mitochondrial fusion machinery. The phosphorylation of Mec1 by Snf1 can be detected by anti-phospho-(Ser/Thr) AMPK substrate (P-S/T2-102) antibody (Yi et al., 2017). Consistent with our previous results, aerobic respiratory deficiency caused by the absence of mitochondrial fusion machinery resulted in the inability of Snf1 to phosphorylate Mec1 under glucose starvation (Figure 3D; Yi et al., 2017). To clarify the underlying molecular mechanism, Snf1 activity was detected under the condition of energy deficiency. As shown in Figures 3E,F, the same as wild type, phosphorylation level of Snf1 under glucose starvation was significantly increased in mitochondrial fusion machinery gene deleted yeast strains, indicating that upon glucose starvation, mitochondrial fusion machinery did not participate in the activation of Snf1. Subsequently, immunoprecipitation assay was carried out for testing Snf1-Mec1 association in full medium and SD-G, the results showed that the interaction between Snf1 and Mec1 significantly decreased in *fzo1*Δ, *ugo1*Δ, and *mgm1*Δ yeast strains in full medium (Figure 3G). Combined with the results above, we concluded that mitochondrial fusion machinery is required for the binding of Snf1 and Mec1 through participating in mitochondrial aerobic respiration, thus regulating the phosphorylation of Snf1 on Mec1, resulting in recruitment of Atg1 and other autophagic proteins to PAS to initiate autophagy under glucose starvation condition.

### Mitochondrial Fusion Machinery Is Required for the Dissociation of Mec1 From Mitochondria During Prolonged Glucose Starvation

**FIGURE 4:**
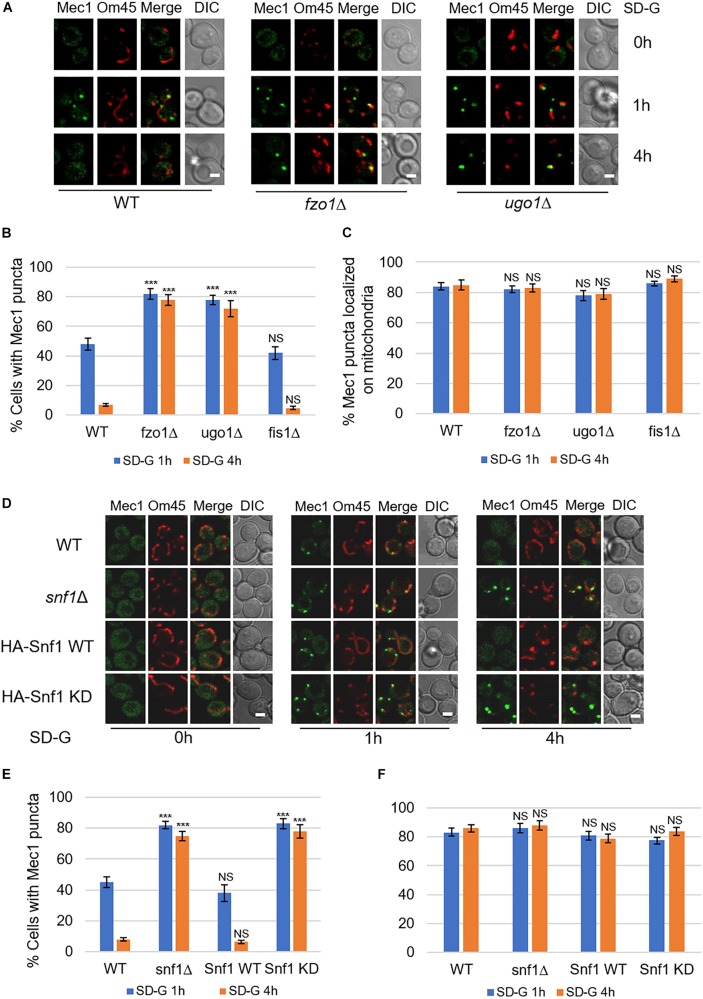
Mitochondrial fusion machinery is required for the dissociation of Mec1 from mitochondria during prolonged starvation. **(A,D)** Co-expression of GFP-Mec1 and Om45-Cherry were expressed in the indicated yeast strains. Cells were cultured in SD-G medium for 0, 1, and 4 h, and then viewed by laser confocal microscopy. Scale bar, 2 μm. **(B,E)** Strains from **(A,D)** were analyzed for the number of cells with Mec1 puncta. *n* = 300 cells pooled from three independent experiments. Data are presented as means ± SD. ***p < 0.001; NS, not significant; two-tailed Student’s *t*-tests were used. **(C,F)** Quantification of mitochondrial GFP-Mec1 puncta in cells from **(A,D)**. Mec1 puncta were examined in 300 cells pooled from three independent experiments. Data are presented as means ± SD. NS, not significant; two-tailed Student’s *t*-tests were used.

In the early stage of glucose starvation, recruitment of Mec1 to mitochondria is crucial for its phosphorylation by Snf1. And with prolonged starvation, Mec1 dissociates from mitochondria (Yi et al., 2017). To explore whether mitochondrial fusion machinery is involved in the dissociation of Mec1 from mitochondria, we knocked out fusion genes *FZO1*, *UGO1*, and fission gene *FIS1* in yeast cells co-expressing mitochondrial marker Om45-Cherry and GFP-Mec1, respectively. Image data showed that upon 4 h of glucose starvation, Mec1 dissociates from mitochondria in wild type and *fis1*Δ yeast cells (Figure 4A and Supplementary Figure S3). In contrast, knockout of *FZO1* and *UGO1* greatly inhibited the dissociation of Mec1 from mitochondria (Figure 4A). Statistical analysis indicated that Mec1 puncta was still mostly associated with mitochondria under prolonged glucose starvation (Figures 4B,C).

Based on the fact that mitochondrial fusion machinery is involved in the phosphorylation of Mec1 by Snf1, we speculated that the kinase activity of Snf1 is important in the dissociation of Mec1 from mitochondria. To test this possibility, we knocked out *SNF1* in the wild-type co-expressing Om45-Cherry and GFP-Mec1 yeast cells, and then re-introduced empty vector, Snf1 wild-type, Snf1 kinase dead plasmid into *snf1*Δ yeast cells. Image data showed that empty vector and Snf1 kinase-dead greatly inhibit the dissociation of Mec1 from mitochondria, while Snf1 wild-type plasmid rescued this phenotype (Figures 4D–F). Thus, the dissociation of Mec1 from mitochondria during prolonged glucose starvation is regulated by the kinase activity of Snf1 and mitochondria fusion machinery.

### Glucose Starvation Does Not Induce Mitophagy

**FIGURE 5:**
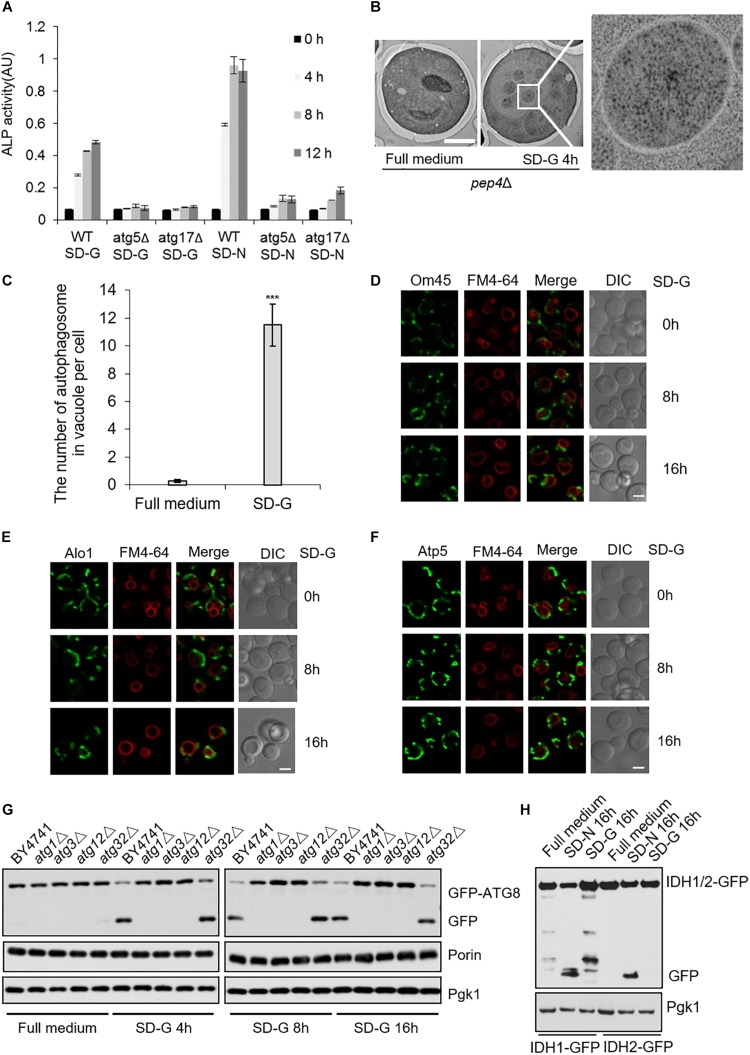
Glucose starvation doesn’t induce mitophagy. **(A)** ALP assay for autophagic activity. In wild type, *atg5*Δ, and *atg17*Δ cells, ALP activity was tested at the indicated time points under SD-G or SD-N conditions from *n* = 3 independent experiments. Error bars indicate standard deviation (SD). **(B)** EM analysis of autophagy in *pep4*Δ cells grown in full medium or SD-G. Scale bar, 2 μm. **(C)** 50 cells from **(B)** were analyzed for the number of autophagosomes inside the vacuole per cell from *n* = 3 independent experiments. Error bars indicate standard deviation (SD). ***p < 0.001; two-tailed Student’s *t*-tests were used. **(D–F)** Cells expressing the GFP-tagged mitochondrial proteins Om45, Alo1, and Atp5 were glucose starved for 0, 8, and 16 h. Cells were viewed by laser confocal microscopy. Scale bar, 2 μm. FM4-64 is a vacuole membrane dye. **(G)** Wild type (BY4741), *atg1*Δ, *atg3*Δ, *atg12*Δ and *atg32*Δ cells expressing GFP-Atg8 were harvested at time point 0 (grown in full medium) and after 4, 8, and 16 h of glucose starvation. The cleavage of GFP-Atg8 and the status of Porin and Pgk1 were measured by western blot using the indicated antibodies. **(H)** Cells expressing the GFP-tagged mitochondrial proteins IDH1 and IDH2 were cultured in SD-G and SD-N for 16 h, respectively, the cleavage of IDH1-GFP and IDH2-GFP were detected by western blot using anti-GFP antibody.

Next, to investigate autophagy level induced by energy deficiency, we compared autophagy under SD-N and SD-G by ALP assay (Araki et al., 2017). As shown in Figure 5A, the ALP activity under nitrogen starvation is about two times higher than that of under glucose starvation, indicating the intensity of autophagy induced by glucose starvation is weaker than that by nitrogen starvation. *ATG5* and *ATG17* knockout completely inhibited the increase of ALP activity, suggesting that Atg5 and Atg17 are essential for glucose starvation-induced autophagy. To clarify the existence of autophagosome in vacuole, electron microscopy experiments were carried out on *pep4*Δ yeast cells under glucose starvation condition (Epple et al., 2001; Kabeya et al., 2009). As shown in Figures 5B,C, there were more than 10 autophagosomes per vacuole after 4 h of glucose starvation, further suggesting that glucose starvation can indeed induce autophagy.

The fusion machinery of mitochondria specifically essential for energy deficiency -induced autophagy prompts us to test whether mitophagy can be induced upon energy deprivation. We selected three mitochondrial proteins: Atp5, mitochondrial inner membrane protein; Om45 and Alo1, mitochondrial outer membrane protein (Yaffe et al., 1989; Boyer, 1997; Sickmann et al., 2003). Image data showed that Om45, Atp5, and Alo1 did not enter the vacuole after 8 and 16 h of glucose starvation (Figures 5D–F and Supplementary Figure S4A). Subsequently, we knocked out autophagy essential genes *ATG1*, *ATG3*, *ATG12*, and mitophagy receptor gene *ATG32* to detect the degradation of endogenous mitochondrial protein Porin. Western blot results showed that in the wild type yeast strain, glucose starvation induces the cleavage of GFP-Atg8, but Porin is not degraded by autophagy at the indicated time point. Inhibition of autophagy and mitophagy did not lead to the accumulation of Porin (Figure 5G and Supplementary Figure S4B). Furthermore, we investigated two GFP-labeled mitochondrial proteins Idh1 and Idh2, which are usually used as the substrate of mitophagy in yeast (Wu and Tu, 2011). Western blot results showed that Idh1-GFP and Idh2-GFP can be cleavaged by nitrogen starvation, not by glucose starvation-induced autophagy (Figure 5H). Taken together, these results indicated that glucose starvation does not induce mitophagy, although mitochondria plays an important role in the initiation of energy deficiency-induced autophagy.

## Discussion

In this study, the relationship between mitochondrial fusion machinery and energy deficiency-induced autophagy has been fully elucidated. Our findings suggested that mitochondrial fusion machinery is essential for energy deficiency-induced autophagy. Deletion of mitochondrial fusion machinery genes significantly impaired the recruitment of autophagy proteins Atg1 and other autophagic proteins to PAS, mitochondrial aerobic respiration, the association of Snf1 with Mec1, phosphorylation of Mec1 by Snf1, and the disassociation of Mec1 from mitochondria. Despite mitochondrial fusion machinery is required for energy deficiency-induced autophagy, glucose starvation did not induce mitophagy. These results strongly suggested that autophagy induced by energy deficiency has its unique signaling pathway and molecular regulatory mechanism.

The fusion, fission and tubulation of mitochondria constitutes a highly dynamic network structure of mitochondria. The morphology of mitochondria is crucial for maintaining its own quality and quantity (Okamoto and Shaw, 2005). Our previous results showed that mitochondrial aerobic respiration is involved in the phosphorylation of Mec1 by Snf1, while phosphorylated Mec1 promoted the binding of Atg1 and Atg13 under glucose starvation (Yi et al., 2017). Oxygen consumption assay showed that only mitochondrial fusion machinery regulated mitochondrial aerobic respiration rate, and thus promotes the phosphorylation of Mec1 by Snf1. To elucidate how mitochondrial respiration regulates the phosphorylation of Mec1 by Snf1, the activation of Snf1 and the association of Mec1 with Snf1 in the absence of glucose was examined. Immunoprecipitation experiment results showed that the absence of mitochondrial fusion machinery genes led to significant decrease in the interaction between Snf1 and Mec1, which affected the phosphorylation of Mec1 by Snf1. In addition, mitochondrial respiration rate remained defective in fission and fusion double mutant albeit with restored mitochondrial morphology, indicating that aerobic respiratory deficiency is independent of mitochondrial morphological changes caused by mitochondrial fusion machinery deletion. These results showed that the mitochondrial fusion machinery involved in the autophagy induced by energy deficiency regulated the phosphorylation of Mec1 by Snf1 by affecting the association of Snf1 with Mec1.

With prolonged glucose starvation, Mec1 dissociated from mitochondria, while this dissociation of Mec1 was inhibited by mitochondrial fusion machinery genes knockout, suggesting Mec1 dissociation from mitochondria is regulated by fusion machinery. To understand the molecular mechanism of Mec1 dissociation, we knocked out *SNF1* gene, and then carried out *SNF1* gene replenishment experiments. The results showed that Mec1 remained associated with mitochondria in the absence of kinase activity of Snf1. As *SNF1* deletion and Snf1 KD inhibited aerobic respiration in glucose starvation (Yi et al., 2017), we speculated that mitochondrial aerobic respiration regulates the dissociation of Mec1 from mitochondria. In future, the phosphorylation substrate of Snf1 which regulated the dissociation of Mec1 from mitochondria need to be identified. In the early stage of glucose starvation, Mec1 was recruited onto mitochondria and then left mitochondria (Yi et al., 2017). In previous study, we found that Mec1 was recruited to mitochondria together with Atg1–Atg13. Based on this fact, we speculated that mitochondria might serve as a protein factor recruitment platform and a source of autophagosome membrane for autophagosome biogenesis under energy deprivation.

Since the fusion machinery of mitochondria is involved in the glucose starvation-induced autophagy, mitophagy was detected under glucose starvation condition. Image and western-blot data showed that glucose starvation does not induce mitophagy. So why energy deficiency does not induce mitophagy? Based on our data, we proposed a hypothesis that under glucose starvation, cells need to provide basic energy to maintain the necessary life activities for survival. As the factory for cells to generate energy, mitochondria need to maintain at certain quantity to provide energy for cell life activities, avoiding being damaged and removed by mitophagy. The phosphorylation modification of mitophagy receptor Atg32 by casein kinase 2 has been reported to be important for the initiation of mitophagy (Kanki et al., 2013). The next step is to test whether Atg32 can be phosphorylated by casein kinase 2 upon glucose starvation, which helps better understand the molecular mechanism of energy deficiency without inducing mitophagy. In summary, our study uncovers mitochondrial fusion machinery specifically essential for glucose starvation-induced autophagy, which provides a perspective for us to understand the biological significance of maintaining mitochondrial aerobic respiratory during energy deficiency.

## Materials and Methods

### Yeast Strains, Constructs and Growth Conditions

All yeast strains and plasmids used in this study are listed in Supplementary Table S1. The related yeast strains were originated from wild type BY4741 and verified by western blot analysis with the indicated antibody or polymerase chain reaction (PCR) (Vazyme, P505-d1) (Janke et al., 2004). All mutant plasmids were sequenced and tested by western blot. Yeast cells were grown at 30°C in corresponding synthetic complete media (0.17% yeast nitrogen base without amino acids and ammonium sulfate, 0.5% ammonium sulfate, 2% dextrose, and 0.5% casamino acids) (rich medium). For autophagy induction, cells grown to mid-log phase in rich medium were subjected to nitrogen starvation medium (SD-N; 0.17% yeast nitrogen base without amino acids and ammonium sulfate, and 2% glucose) or glucose starvation medium (SD-G; 0.17% yeast nitrogen base without amino acids and ammonium sulfate, 0.5% ammonium sulfate, and 0.5% casamino acids) for 4 h at 30°C.

### Antibodies

The company, product number and dilution ratio of antibody in this study are as follows: anti-GFP (Roche, 11814460001, 1:2500), anti-FLAG (Sigma, F1804, 1:2500), anti-HA (Abmart, M20003L, 1:3000), anti-phospho-AMPKα(Thr172) (Cell Signaling Technology, 2535S, 1:1000), anti-Phospho-(Ser/Thr) AMPK Substrate (Cell Signaling Technology, 5759, 1:1000), anti-Porin (Thermo Fisher Scientific, A6449, 1:10000), anti-PGK1 (Nordic Immunology, NE130/7S, 1:10000), Goat anti-Mouse IgG1, Human ads-HRP (SouthernBiotech, 1070-05, 1:10000), Goat anti-Rabbit, Human ads-HRP (SouthernBiotech, 4010-05, 1:10000).

### Microscopy, Western Blots, and Immunoprecipitation

The yeast strains with different fluorescent tags grew to the log-growth phase, and then were treated with the indicated conditions. The cells were observed by fluorescence inversion microscope (IX83; Olympus) or laser confocal microscope (FV1000; Olympus) at the indicated time. Images were processed in ImageJ software and Adobe Photoshop. The images were not manipulated except brightness and contrast adjustments. Yeast protein extraction, western blotting and immunoprecipitation in this study were carried out according to the previously described methods (Yi et al., 2017).

### Determination of Oxygen Consumption of Cells

Yeast cells were grown to log-growth phase, and then were subjected to glucose starvation or nitrogen starvation for 30 min. Oxygen consumption was measured using Oxygraph-2k (O2K, OROBOROS Instruments, Innsbruck, Austria) (Makrecka-Kuka et al., 2015).

### Electron Microscopy

*pep4*Δ yeast strain was grown to log-growth phase, and then were subjected to glucose starvation for 0 and 4 h. Yeast was centrifuged using horizontal rotor centrifuge at 2,000 rpm for 2 min, then the supernatant was removed. Yeast was frozen by high-pressure freezer (Leica EM HPM100) with 100 μm deep carriers and 1-hexadecene as cryoprotect. Freeze substitution was performed by using a Leica EM AFS2 in dry acetone containing 5% ddH_2_O, 1% OsO_4_, and 0.1% uranyl acetate over a long period as follows: −90°C for 72 h, 2°C per hour increase for 15 h, −60°C for 8 h, 2°C per hour increase for 15 h, and −30°C for 8 h. After 1 h on ice, samples were then washed three times in pure acetone on ice, infiltrated in 1:1 (resin/acetone) 12 h, 2:1 12 h, 3:1 12 h, resin 12 h, resin 1 h Pon 812 resin and embedded. The polymerization was performed at 60°C for 48 h. Ultrathin sections were made using an ultramicrotome (Leica EM UC7), post-stained in uranyl acetate for 30 min and in lead citrate for 5 min. Grids were imaged with a transmission electron microscope (H-7650B; Hitachi) (Takeshige et al., 1992).

### ALP Assay

The indicated yeast strains were grown to log-growth phase, and then were starved in nitrogen starvation and glucose starvation medium for 0, 4, 8, and 12 h. ALP assay was carried out as described previously (Araki et al., 2017).

### Quantification and Statistical Analysis

For all quantitative and statistical analyses, the mean values are displayed together with the standard deviation (SD, shown as error bars). The phosphorylation level of Snf1 was quantified by Image J software. Student’s two-tailed *t*-test was performed for *p*-value calculations according to all comparisons between control and experiment conditions.

## Data Availability Statement

The datasets generated for this study are available on request to the corresponding author.

## Author Contributions

CW, WY, ZH, and CY conceived the experiments. ZH and CY wrote the manuscript and supervised the project. CW, WY, WK, WL, WW, SL, YC, LW, YL, JT, and LZ carried out the experiments. All authors discussed the manuscript and contributed to preparing the manuscript.

## Conflict of Interest

The authors declare that the research was conducted in the absence of any commercial or financial relationships that could be construed as a potential conflict of interest.
