# Supplementary material for: Mitochondrial Fusion Machinery Specifically Involved in Energy Deprivation-Induced Autophagy
Source: Front Cell Dev Biol. 2020 Apr 7;8:221. doi: 10.3389/fcell.2020.00221 (PMC7154291; doi:10.3389/fcell.2020.00221)
Supplement: Supplementary file 1 [file Presentation_1.pdf]

Supplementary Materials

Supplementary Figures

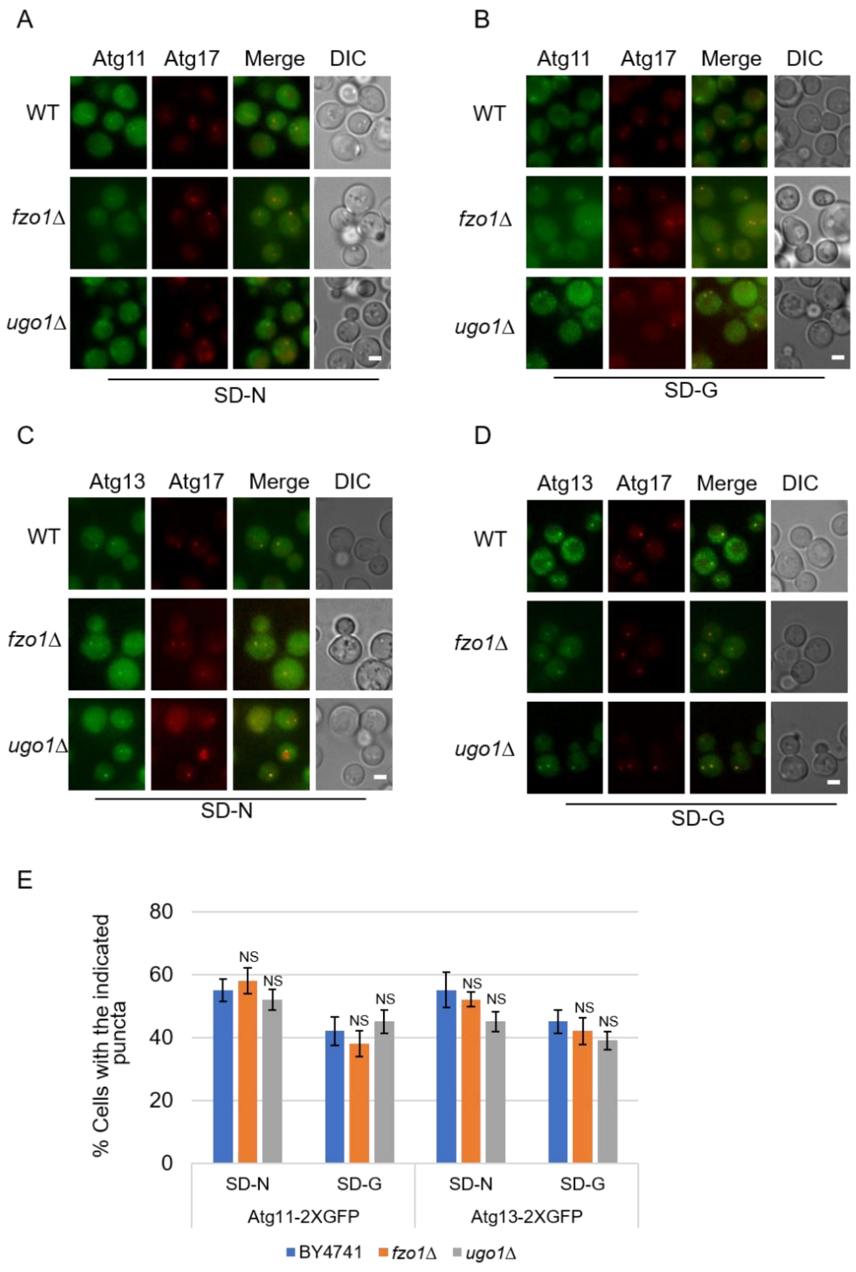

Figure S1. The deletion of mitochondrial fusion machinery did not affect the recruitment of Atg11 and Atg13 protein to PAS under glucose starvation condition. (A-D) Atg11-2XGFP and Atg17-2XCherry or Atg13-2XGFP and Atg17-2XCherry were co-expressed in the wild type (WT), *fzo1*Δ, and *ugo1*Δ yeast strains. Yeast cells were cultured in SD-N and SD-G for 1 h. Cells were imaged by inverted fluorescence

microscope. Scale bar, 2 µm. **(E)** Strains from (A-D) were analyzed for the number of cells with the indicated puncta. n= 300 cells pooled from 3 independent experiments. Data are presented as means ± s.d. NS, not significant; two-tailed Student's t tests were used.

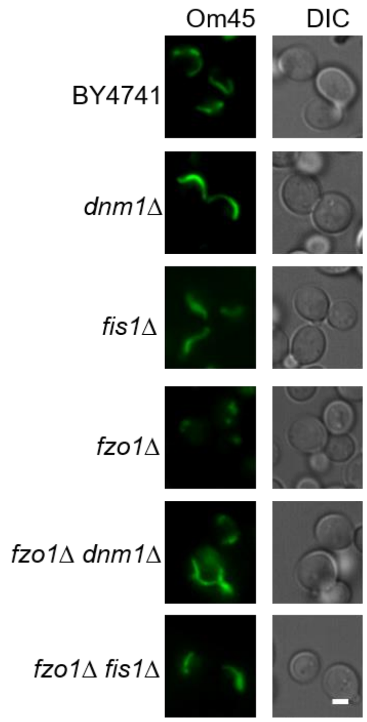

Figure S2. Mitochondrial morphology is restored in fission and fusion double mutant cells. Om45-GFP (mitochondria marker) was expressed in WT (BY4741), *dnm1*Δ, *fis1*Δ, *fzo1*Δ, *fzo1*Δ *dnm1*Δ, and *fzo1*Δ *fis1*Δ strains. Yeast cells were grown to the log-growth phase. Cells were imaged by inverted fluorescence microscope. Scale bar, 2 µm.

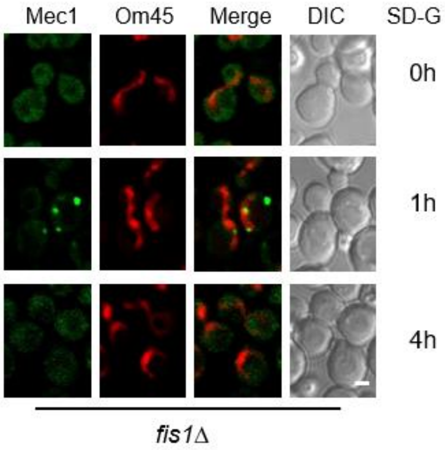

Figure S3. Fis1 is not involved in the dissociation of Mec1 from mitochondria under prolonged glucose starvation. GFP-Mec1 and Om45-Cherry were co-expressed in *fis1*Δ yeast strain. Yeast cells were grown to the log-growth phase, then subjected to SD-G for 0 h, 1 h and 4 h. Cells were viewed by confocal laser scanning microscope. Scale bar, 2 μm.

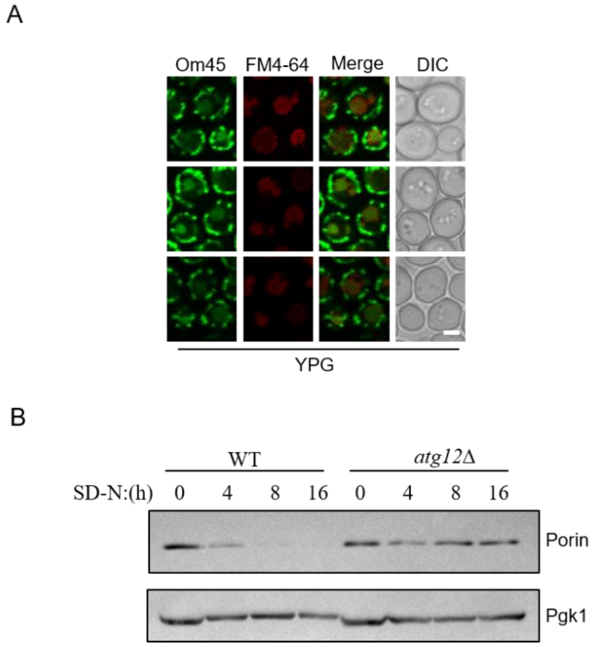

Figure S4. Mitophagy in YPG medium and nitrogen starvation condition. (A) Cells expressing Om45-GFP, Alo1-GFP or Atp5-GFP were grown to log-growth phase in SD-His medium, and then subjected to YPG medium for 24 h. Cells were viewed by

confocal microscope. Scale bar, 2 µm. **(B)** Wild type cells were grown to log-growth phase in YPD medium, and then starved in SD-N medium for 0 h, 4 h, 8 h and 16 h. Porin and Pgk1 proteins were measured by western blot using the corresponding antibodies.

Supplementary Tables

Table S1. Yeast strains in this study

Name	Genotype	Source
YM1	*BY4741*	Lab stock
YM2	*BY4741 fis1Δ::KanMX6 pRS316[GFP-Atg8 Ura3]*	In this study
YM3	*BY4741 dnm1Δ::KanMX6 pRS316[GFP-Atg8 Ura3]*	In this study
YM4	*BY4741 caf4Δ::KanMX6 pRS316[GFP-Atg8 Ura3]*	In this study
YM5	*BY4741 mdv1Δ::KanMX6 pRS316[GFP-Atg8 Ura3]*	In this study
YM6	*BY4741 mgm1Δ::KanMX6 pRS316[GFP-Atg8 Ura3]*	In this study
YM7	*BY4741 pcp1Δ::KanMX6 pRS316[GFP-Atg8 Ura3]*	In this study
YM8	*BY4741 ugo1Δ::KanMX6 pRS316[GFP-Atg8 Ura3]*	In this study
YM9	*BY4741 fzo1Δ::KanMX6 pRS316[GFP-Atg8 Ura3]*	In this study
YM10	*BY4741 mdm20Δ::KanMX6 pRS316[GFP-Atg8 Ura3]*	In this study
YM11	*BY4741 mdm31Δ::KanMX6 pRS316[GFP-Atg8 Ura3]*	In this study
YM12	*BY4741 mdm32Δ::KanMX6 pRS316[GFP-Atg8 Ura3]*	In this study
YM13	*BY4741 mdm33Δ::KanMX6 pRS316[GFP-Atg8 Ura3]*	In this study
YM14	*BY4741 gem1Δ::KanMX6 pRS316[GFP-Atg8 Ura3]*	In this study
YM15	*BY4741 Atg17-2XCherry::hphNT1 Atg2-2XGFP::Ura3*	in this study
YM16	*BY4741 fzo1Δ::KanMX6 Atg17-2XCherry::hphNT1 Atg2-2XGFP::Ura3*	in this study
YM17	*BY4741 ugo1Δ::KanMX6 Atg17-2XCherry::hphNT1 Atg2-2XGFP::Ura3*	in this study
YM18	*BY4741 Atg17-2XCherry::hphNT1 Atg5-2XGFP::Ura3*	in this study
YM19	*BY4741 fzo1Δ::KanMX6 Atg17-2XCherry::hphNT1 Atg5-2XGFP::Ura3*	in this study
YM20	*BY4741 ugo1Δ::KanMX6 Atg17-2XCherry::hphNT1 Atg5-2XGFP::Ura3*	in this study
YM21	*BY4741 Atg17-2XCherry::hphNT1 Atg11-2XGFP::Ura3*	in this study
YM22	*BY4741 fzo1Δ::KanMX6 Atg17-2XCherry::hphNT1 Atg11-2XGFP::Ura3*	in this study
YM23	*BY4741 ugo1Δ::KanMX6 Atg17-2XCherry::hphNT1 Atg11-2XGFP::Ura3*	in this study
YM24	*BY4741 Atg17-2XCherry::hphNT1 pRS316[GFP-Atg1 Ura3]*	in this study
YM25	*BY4741 fzo1Δ::KanMX6 Atg17-2XCherry::hphNT1 pRS316[GFP-Atg1 Ura3]*	in this study
YM26	*BY4741 ugo1Δ::KanMX6 Atg17-2XCherry::hphNT1 pRS316[GFP-Atg1 Ura3]*	in this study
YM27	*BY4741 3XFLAG-Mec1::natNT2 pRS316[HA-Snf1 Ura3]*	in this study
YM28	*BY4741 fzo1Δ::KanMX6 3XFLAG-Mec1::natNT2 pRS316[HA-Snf1 Ura3]*	in this study

YM29	*BY4741 mgm1Δ::KanMX6 3XFLAG-Mec1::natNT2 pRS316[HA-Snf1 Ura3]*	in this study
YM30	*BY4741 ugo1Δ::KanMX6 3XFLAG-Mec1::natNT2 pRS316[HA-Snf1 Ura3]*	in this study
YM31	*BY4741 GFP-Mec1::natNT2 Om45-Cherry::KanMX6*	Lab stock
YM32	*BY4741 fzo1Δ::KanMX6 GFP-Mec1::natNT2 Om45-Cherry::KanMX6*	In this study
YM33	*BY4741 ugo1Δ::KanMX6 GFP-Mec1::natNT2 Om45-Cherry::KanMX6*	In this study
YM34	*BY4741 fis1Δ::KanMX6 GFP-Mec1::natNT2 Om45-Cherry::KanMX6*	In this study
YM35	*BY4741 snf1Δ::KanMX6 GFP-Mec1::natNT2 Om45-Cherry::KanMX6 pRS316*	In this study
YM36	*BY4741 snf1Δ::KanMX6 GFP-Mec1::natNT2 Om45-Cherry::KanMX6 pRS316[HA-Snf1 Ura3]*	In this study
YM37	*BY4741 snf1Δ::KanMX6 GFP-Mec1::natNT2 Om45-Cherry::KanMX6 pRS316[HA-Snf1 KD Ura3]*	In this study
YM38	*BY4741 pho8::pho8Δ60 pho13Δ:: hphNT1*	Lab stock
YM39	*BY4741 pho8::pho8Δ60 pho13Δ:: hphNT1 atg5Δ::KanMX6*	Lab stock
YM40	*BY4741 pho8::pho8Δ60 pho13Δ:: hphNT1 atg17Δ::KanMX6*	Lab stock
YM41	*BY4741 pep4Δ::KanMX6*	Lab stock
YM42	*BY4741 Om45-GFP::HisMX6*	Lab stock
YM43	*BY4741 Alo1-GFP::HisMX6*	Lab stock
YM44	*BY4741 Atp5-GFP::HisMX6*	Lab stock
YM45	*BY4741 atg1Δ::KanMX6 pRS316[GFP-Atg8 Ura3]*	Lab stock
YM46	*BY4741 atg3Δ::KanMX6 pRS316[GFP-Atg8 Ura3]*	Lab stock
YM47	*BY4741 atg12Δ::KanMX6 pRS316[GFP-Atg8 Ura3]*	Lab stock
YM48	*BY4741 atg32Δ::KanMX6 pRS316[GFP-Atg8 Ura3]*	Lab stock
YM49	*BY4741 IDH1-GFP::HisMX6*	Lab stock
YM50	*BY4741 IDH2-GFP::HisMX6*	Lab stock
YM51	*BY4741 Om45-GFP::HisMX6 dnm1Δ::KanMX6*	In this study
YM52	*BY4741 Om45-GFP::HisMX6 fis1Δ::KanMX6*	In this study
YM53	*BY4741 Om45-GFP::HisMX6 fzo1Δ::KanMX6*	In this study
YM54	*BY4741 Om45-GFP::HisMX6 fzo1Δ::KanMX6 dnm1Δ:: hphNT1*	In this study
YM55	*BY4741 Om45-GFP::HisMX6 fzo1Δ::KanMX6 fis1Δ:: hphNT1*	In this study
YM56	*BY4741 fzo1Δ::KanMX6 fis1Δ:: hphNT1 pRS316[GFP-Atg8 Ura3]*	In this study
YM57	*BY4741 fzo1Δ::KanMX6 dnm1Δ:: hphNT1 pRS316[GFP-Atg8 Ura3]*	In this study
